# Three Output Membrane Hydrocyclone: Classification and Filtration

**DOI:** 10.3390/molecules24061116

**Published:** 2019-03-21

**Authors:** Jhao-Yi Lin, Rome-Ming Wu

**Affiliations:** Department of Chemical and Materials Engineering, Tamkang University, 151 Ying-chuan Road, Tamsui, Taipei 25137, Taiwan; jjacky801226tw@gmail.com

**Keywords:** classification, filtration, membrane, hydrocyclone, three outputs

## Abstract

In this study, through simulation and experimental verification, we proposed a novel hydrocyclone in which a tubular ceramic membrane passed through the overflow outlet to the underflow outlet. The centers of overflow and underflow outlets were tubular membranes equipped with an exit of outside-in filtration, and the overflow the underflow outlets were shaped into annular (donut shape) exits. Thus, this novel hydrocyclone has three outlets, namely the overflow dilute liquid, the underflow concentrated liquid, and clear filtrate. This system enabled higher dilution of hydrocyclone overflow concentration than that in the traditional system. Furthermore, underflow was more concentrated, and we obtained a clear filtrate. Therefore, this device can simultaneously perform classification and filtration, which is valuable for special liquid recycling. For instance, in wafer cutting fluid recovery in solar energy processes, the fluid with more silicon can function as the overflow, the fluid with more silicon carbide can function as the underflow, and the polyethylene glycol (PEG) organic solvent can function as the clear filtrate.

## 1. Introduction

Among the classifying equipment [1,2] currently available, hydrocyclones are one of the most popular pieces of equipment [3,4,5] and can be applied in minering [6] and bio-engineering [7,8] processes and slurry treatment [9] (an emerging field). Based on previous studies, hydrocyclones have proven to be universal devices with various advantages such as its compact size, no moving parts, simple and inexpensive manufacturing, and minimal maintenance.

In the past, several attempts have been made to design hydrocyclones with different structures and changing fluid characteristics, with special emphasis on the development of high separation efficiency hydrocyclones. For example, Yoshida et al. [10] developed an electric field-assisted cyclone capable of removing ultrafine particles (0.3 μm) with enhanced efficiency. Hwang and Chou [11] designed a vortex finder structure for improving hydrocyclone particle separation efficiency. Vieira et al. [12,13] proposed a filtering hydrocyclone, in which the conical portion was replaced by the filter material, which enhanced classification efficiency. Because of advances in computing technology [14], computational fluid dynamics (CFD) is being increasingly used in multiphase flow fields [11,15,16,17,18]. Hydrocyclone separator flow pattern complexity, changing hydrocyclone air core patterns, and unsteady flow have been discussed in previous studies. Large-eddy simulation (LES) has been used to simulate cyclone swirls [19,20,21]. LES is a dynamic simulation, and under the same structure and size, LES requires finer grid than any other simulation model. As long as the grid is sufficiently fine, the disturbance within the hydrocyclone flow field coordination can be clearly calculated through LES. Slack et al. [22] used LES to simulate cyclones and observed excellent velocity prediction. The LES turbulence model is an extension of the multiphase flow CFD model that includes the stream of hydrocyclone particles; this model may be a very effective tool to study the effect of the structure size on separation. Most importantly, the hydrocyclones that are replaced by various structures and sizes can be promptly examined. Today, not only does the overall behavior of the flow pattern inside the hydrocyclone prompt the universal application of the technology, but so do many numerical, CFD calculation, and experimental verification research [23,24,25]. Furthermore, the particle–fluid interaction force has been studied [26]. Relatively fewer studies have mentioned the effect of particle concentration. Two fluid numerical simulation models developed recently have shown the influence of particle concentration on hydrocyclone size; in smaller hydrocyclones, more fine particles flow out from the underflow, whereas in larger hydrocyclones, more coarse particles flow out from the overflow [15]. Velocity profiles demonstrated that the turbulence intensity in the vicinity of the air core and in the hydrocyclone inner wall was the highest and those in other locations were diminished [27]. At the spiral flow area under the overflow pipe, the radial pressure gradient, pressure disturbances, as well as the relative pressure disturbance were considerable. That is, the energy loss and turbulent energy dissipation in this region is severe [28]. Thus, controlling the central region of the hydrocyclone or controlling the turbulent structure at the spiral flow area is a highly effective method to improve performance [29].

Membrane filtration and centrifugal driving force have been demonstrated as efficient separation techniques to obtain valuable materials [30,31]. Thus, in this study, we inserted a tubular ceramic membrane in the central region of the hydrocyclone to offset the air core and change the turbulence structure for enhancing separation efficiency; when the tubular membrane was used, this hydrocyclone had one inlet and three outlets [32]. The three outlets are overflow, underflow, and filtrate, which satisfy classification and filtration requirements. The numerical solution can be implemented using commercial CFD FLUENT 6.1 software. The previously mentioned LES model implements turbulence simulation. After the flow field within the hydrocyclone was solved, the motion of the particle flow was tracked using the discrete phase model (DPM) in a Lagrangian reference frame. The CFD method can predict hydrocyclone performance under various operating conditions, and this has been confirmed in the literature [20,33,34]. In addition, the tubular membrane was compared by replacing it with a solid cylindrical tube. Traditional hydrocyclone is labeled THC; the hydrocyclone in which a solid cylinder is inserted in the center is labeled SHC; and the hydrocyclone with a tubular ceramic membrane inserted in the center is labeled MHC.

## 2. Results and Discussion

### 2.1. Volume Fraction of Air and Velocity Distribution

Many studies have indicated that the central air core and the adjacent part of the flow are a forced vortex [28]; the main vortex turbulence energy consumption within the vortex occurs because of energy transfer and friction losses. When the diameter of the overflow increases, the diameter of air core increases, therefore, large eddy crushing devices [29] can reduce energy loss. Between the inner wall of the hydrocyclone and the overflow pipe, more than one area exists at which the direction of the fluid motion is reversed [35]. The maximum tangential direction speed is usually located inside the diameter position of the overflow pipe.

**Figure 1:**
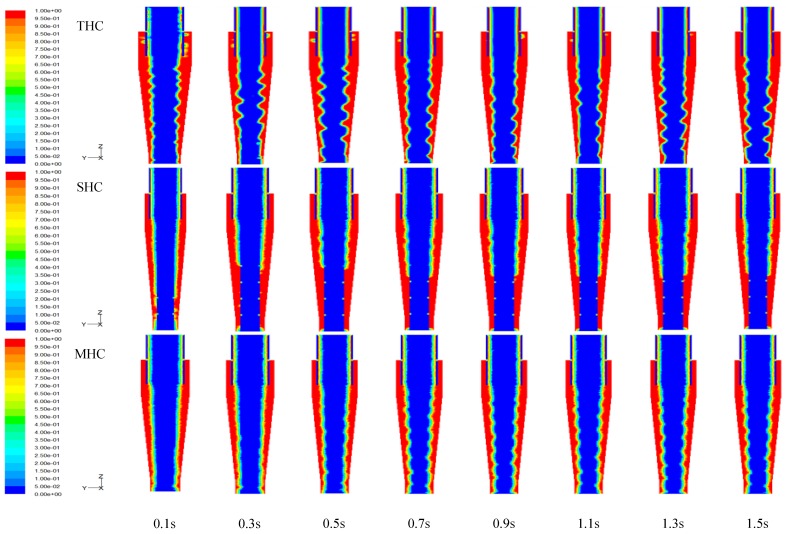
Transient development of air core structure in the hydrocyclone separator system obtained from CFD simulations.

Figure 1 depicts an overtime air core development chart of the three hydrocyclone patterns in this study. From the chart SHC, because of a solid cylinder, the amount of air volume should be minimal. However, some air still exists around the solid cylinder, which results in an overall maximum diameter of the air core. The THC air core is relatively unstable compared to the MHC air core. Therefore, MHC should be able to offset turbulent energy consumption of the central air core.

**Figure 2:**
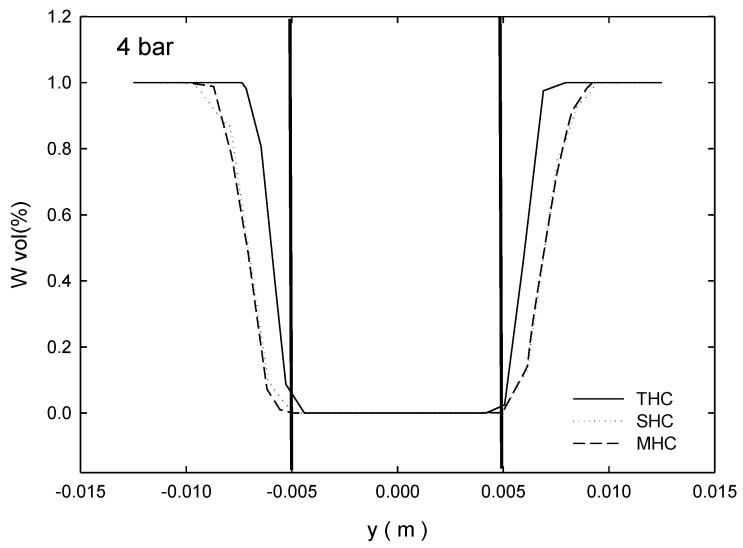
Water volume fraction at Z = 0.125 m.

Figure 2 depicts an XY plane below the overflow pipe bottom (z = 0.125 m, the position of the overflow entrance) and the distribution liquid volume in the radial direction. The diameter of the air core was approximately 14–15 mm. SHC and MHC centers contain a metal tube and a ceramic tubular membrane to block the original air core area. However, an air core is still generated. In addition, the MHC air core was in the range between 6 and −6 mm. Our inference from the figure was that when the MHC performed outside-in filtration, it functioned not only as a simple cross-flow filtration but also as a doping sweep gas–liquid filtration. As a result, the filtration rate was enhanced. In the future, this filtration should be studied further.

**Figure 3:**
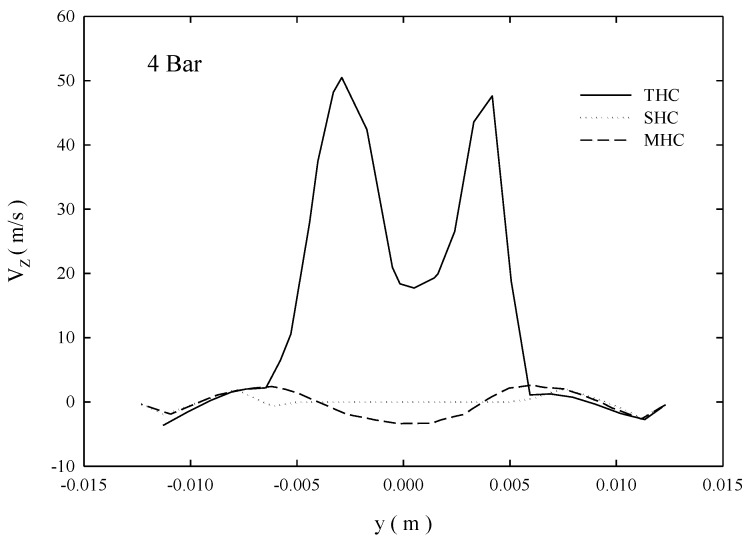
Vertical velocity at Z = 0.125 m.

Figure 3 depicts the Z-axis speed distribution of the XY plane below the overflow pipe bottom (z = 0.125 m). The figure shows that the flow rate of the THC at the air core region was considerably higher than those of SHC and MHC. Cilliers and Harrison [36] indicated that reducing the flow vicinity of the overflow outlet reduced the short circuit flow. Because the central location of the tubular ceramic membrane can offset turbulence energy consumption, MHC should be able to reduce the short circuit flow.

### 2.2. The Concentration and the Filtering Effect of the MHC

**Figure 4:**
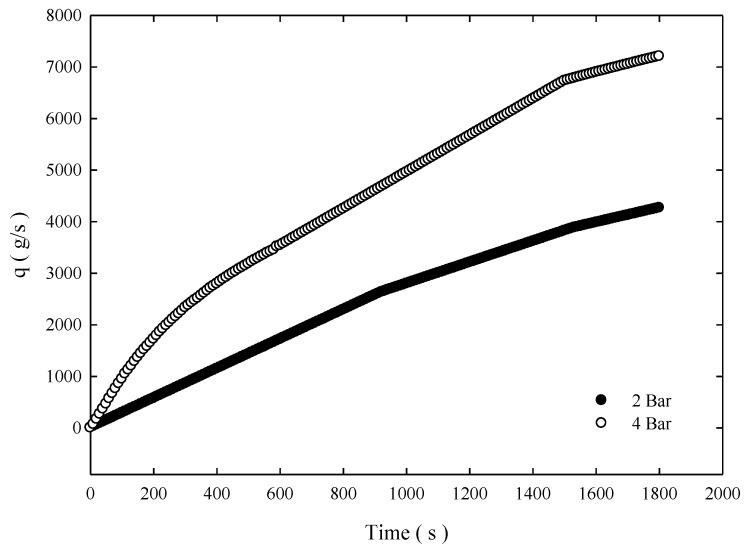
Filtrate obtained for MHC.

MHC is effective in filtration. Figure 4 depicts the filtration rate of MHC at various pressures. The average filtrate can be obtained at approximately 400–450 L/m^2^-h-bar. Because of the MHC material’s effect of reducing turbulent flow and filtering, the concentration difference between overflow and underflow is the largest and benefits the subsequent drying process. As presented in Table 1, the raw material concentration was 0.35%. For THC, the concentrations from overflow and underflow were 0.30% and 0.42%, respectively; For SHC, the concentrations from overflow and underflow were 0.24% and 0.59%, respectively; however, by using MHC, the concentrations from overflow and underflow were 0.21% and 1.31%, respectively.

### 2.3. Particle Trajectories

**Figure 5:**
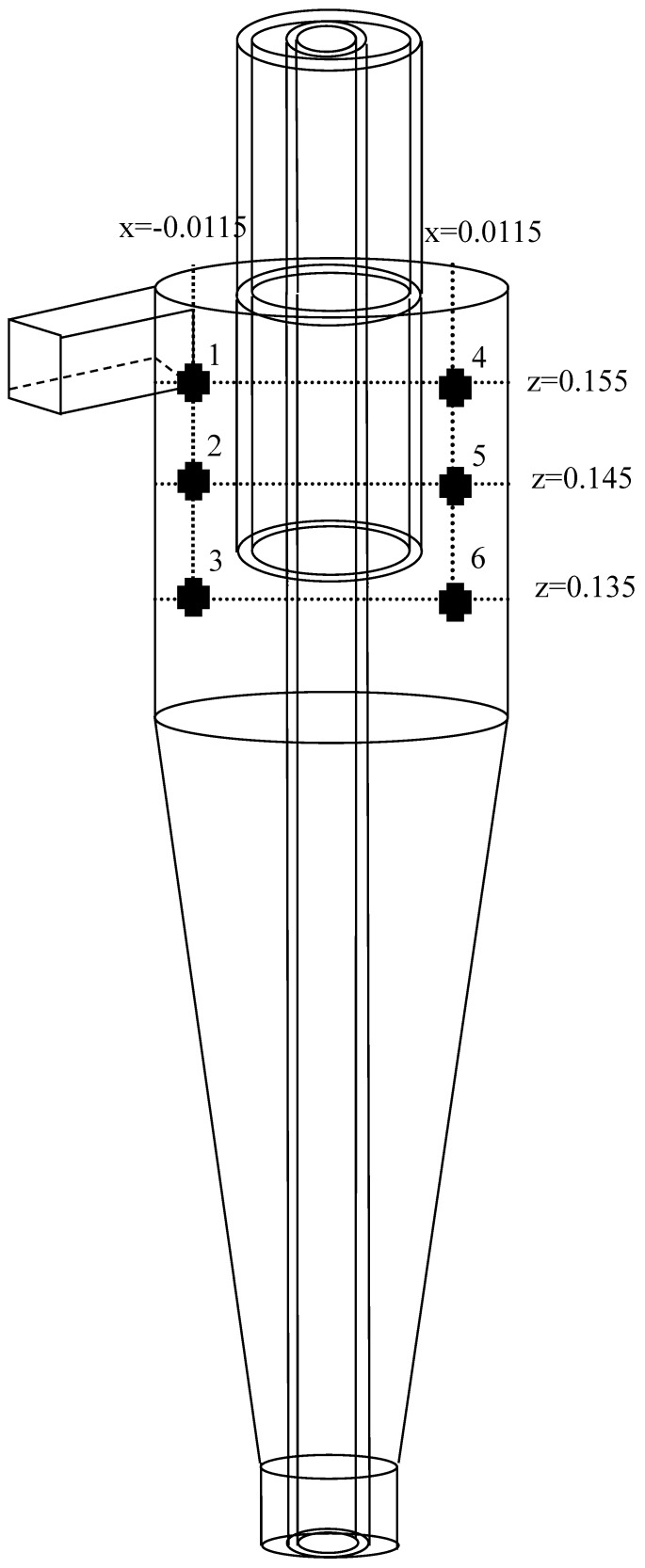
Six release positions in the hydrocyclone.

**Figure 6:**
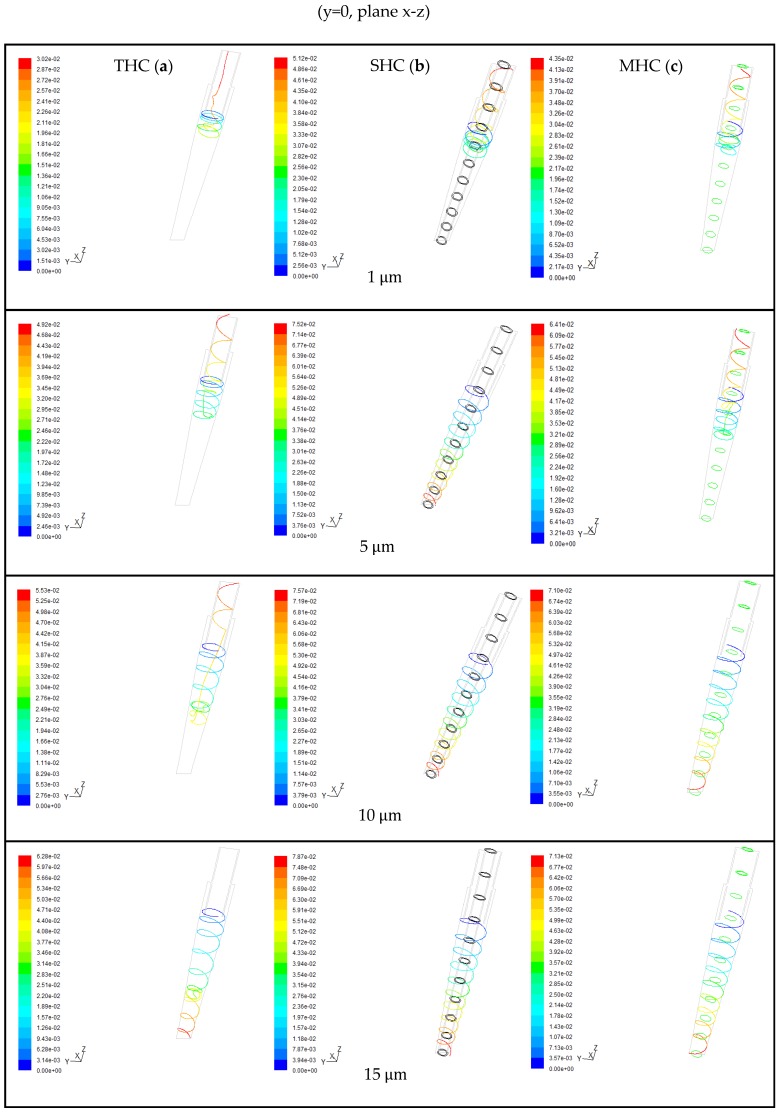
Particle trajectory of size 1, 5, 10, and 15 μm in three hydrocyclones. (**a**) THC; (**b**) SHC; and (**c**) MHC.

**Figure 7:**
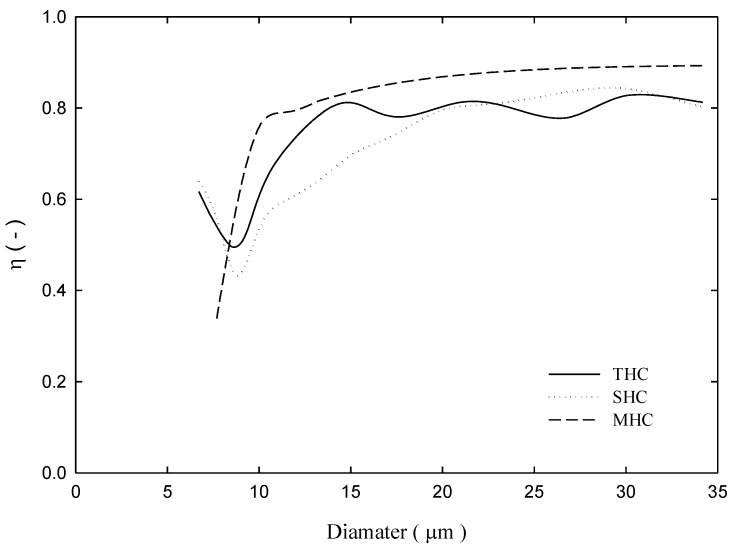
Separation efficiency curve for three hydrocyclones under an inlet pressure of 4 bar.

To further understand the particle trajectory in the hydrocyclone, particularly when the particles are released in the vicinity of the overflow, and to determine whether they ultimately left from overflow or underflow, Figure 5 presents the six release positions of the particles in the study. The horizontal position shows intermediate (X = ±0.0115 m) between the water cyclone overflow tube and the wall. The vertical position shows Z = 0.135, 0.145, 0.155 m. The particle density was set to 3200 kg/m^3^, and the particle sizes were set separately at 1, 5, 10, and 15 μm. According to the simulation result, position 3 of the particle track was slightly different, and those at other positions were similar. Thus, Figure 6 presents the particle trajectory of THC, SHC, and MHC at position 3. For THC, particles of 1, 5, and 10 μm flowed to the overflow outlet, and only 15 μm particles flowed to underflow, which indicates short circuit flow at position 3. Figure 7 indicates that the *d*_50_ for the three hydrocyclones is 8.5 μm. For SHC, only 1 μm particles exited from the overflow. This can be attributed to the solid cylinder inside the hydrocyclone, which causes boundary layer variance. For MHC, particles larger than 10 μm flowed to the underflow.

### 2.4. Classification Efficiency

**Figure 8:**
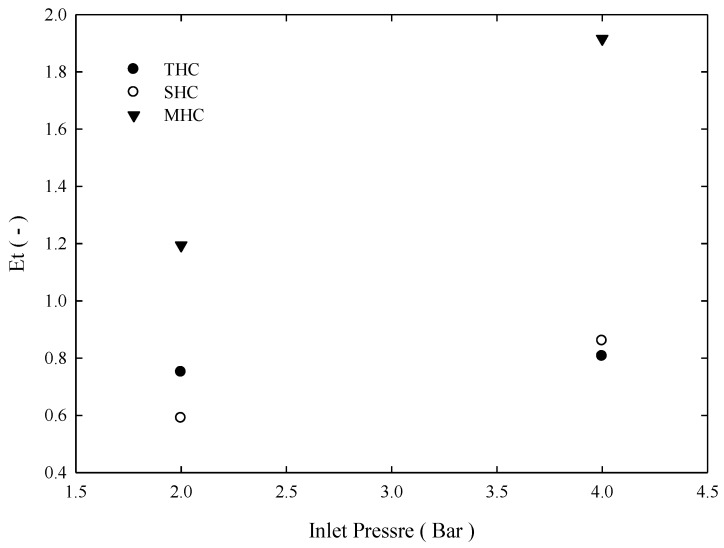
Overall separation efficiency for three hydrocyclones under different inlet pressures.

The classification efficiency curve is the rate of a particle of a certain size flowing from the feeding inlet of the hydrocyclone and leaving from underflow of the hydrocyclone. Figure 7 depicts the classification efficiency curve of the three separators under the 4-bar operation. Under the same feeding conditions, although three hydrocyclones *d*_50_ values were approximately 8.5 μm, the result indicates that a fish hook effect occurs in SHCs and THCs, which indicates that placing a solid cylinder cannot effectively solve the fish hook effect problem. However, for MHCs, the membrane’s permeability eliminates excessive turbulence. Thus, the fish hook effect does not occur and effective separation is obtained. Approximately 80% of >10 μm particles flow out from the underflow outlet. As for THC and SHC, 80% particles flowing from the underflow are approximately larger than 15 and 20 μm. Overall separation efficiency was $E_{t}=(Q_{u}C_{u})/(Q_{f}C_{f})$, and we can see from Figure 8 that irrespective of 4 or 2 bar pressure, the overall separation efficiency of MHC was considerably higher than the other two hydrocyclones.

## 3. Materials and Methods

### 3.1. Materials

**Figure 9:**
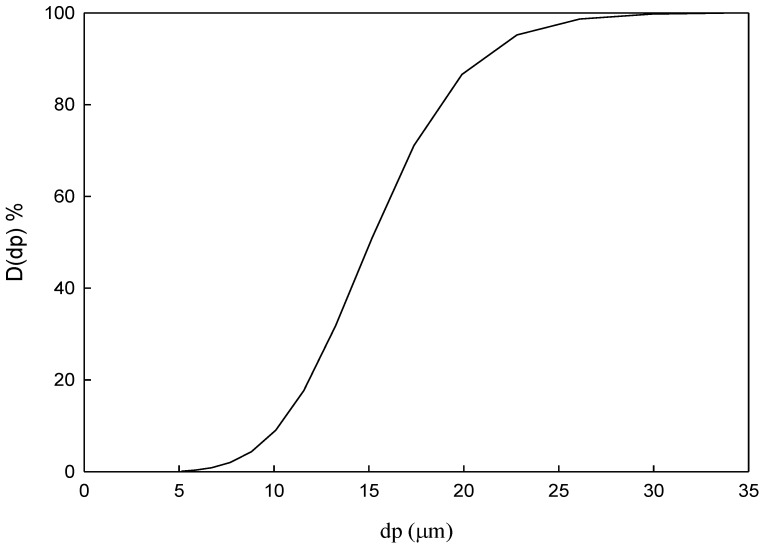
Silicon carbide powder cumulative size distribution used in the experiments.

Silicon carbide powder of 13–23 μm diameter was used in this study. The particle size distribution is presented in Figure 9. The average particle diameter was 16.5 μm, and the specific gravity of particles was 3.2. The adopted composite ceramic tubular membrane was made by TAMI Industries (Nyons, Drôme, France) [http://www.tami-industries.com/en/enadvanced-ceramic-filtration/]. The support layer of the tubular membrane was made of titania, and the filter layer was made of titania and zirconia, with a pore size of 0.8 μm. The tubular membrane diameter and length were 10 mm and 600 mm, respectively. Furthermore, the membrane was single-channel with a surface area of 0.008 m^2^. Laser particle size distribution analyzer model No. Horibala LA-30 (Horiba, Kyoto, Japan) was used to measure particle size distribution from 0.1–600 μm. Based on the light scattering theory, a 405 nm helium-neon LED light source was used to measure the sample particle size distribution on the overflow and underflow.

### 3.2. Experimental Setups and Operation

**Figure 10:**
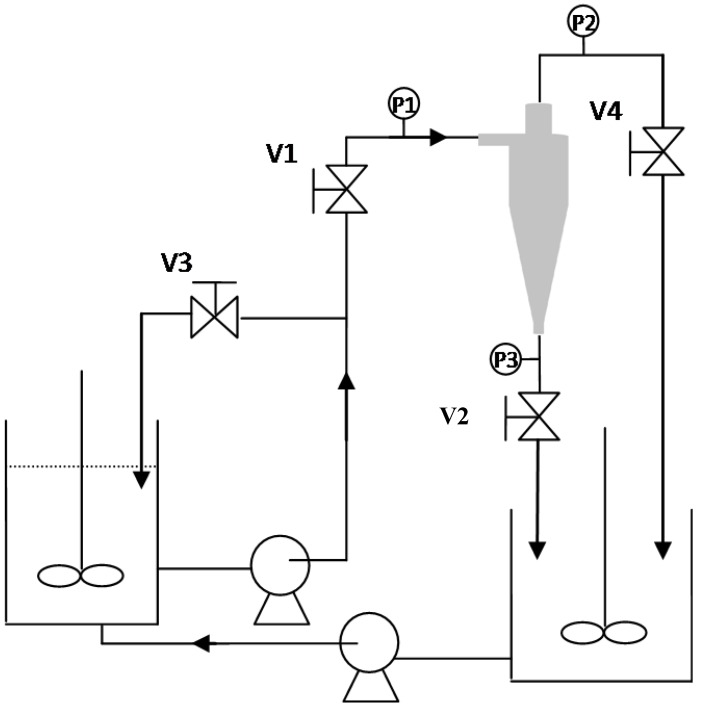
Hydrocyclone separator system experimental setup. The various components include a centrifugal pump, storage tank, agitator, pressure gauges (P1, P2, P3), recycle valve (V3), inlet valve (V1), overflow valve (V4), underflow valve (V2), and the hydrocyclone separator.

The experimental apparatus in this study is presented in Figure 10. First, the bypass valve (V3) was fully opened. Then, the overflow valve (V4) and underflow valve (V2) were fully opened, the feeding end valve (V1) was adjusted, and the feeding gauge pressure was observed (P1) until the pressure reached the desired level. The inlet pressure, overflow pressure (P2), and underflow pressure (P3) were recorded. The diluted liquid from overflow and the concentrated liquid stream from underflow were measured and sampled, and the amount of clear liquid filtrate was recorded at the outlet end of the outside-in filtration. Particle distribution, concentration, and separation efficiency were then obtained.

### 3.3. Geometry and Meshes

**Figure 11:**
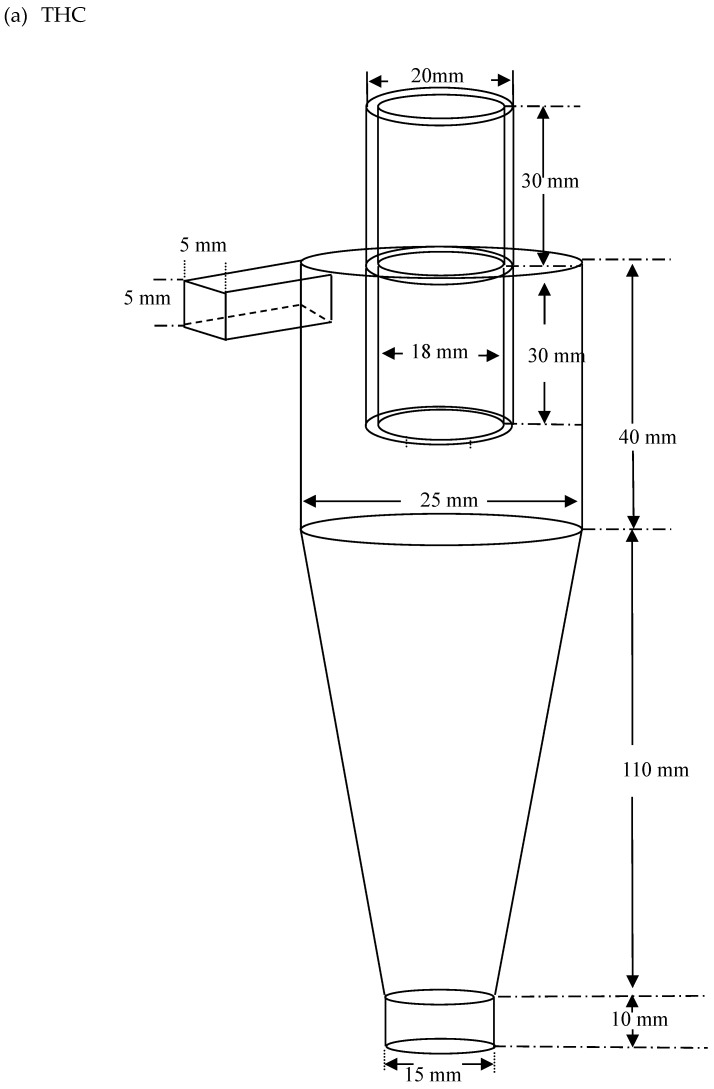

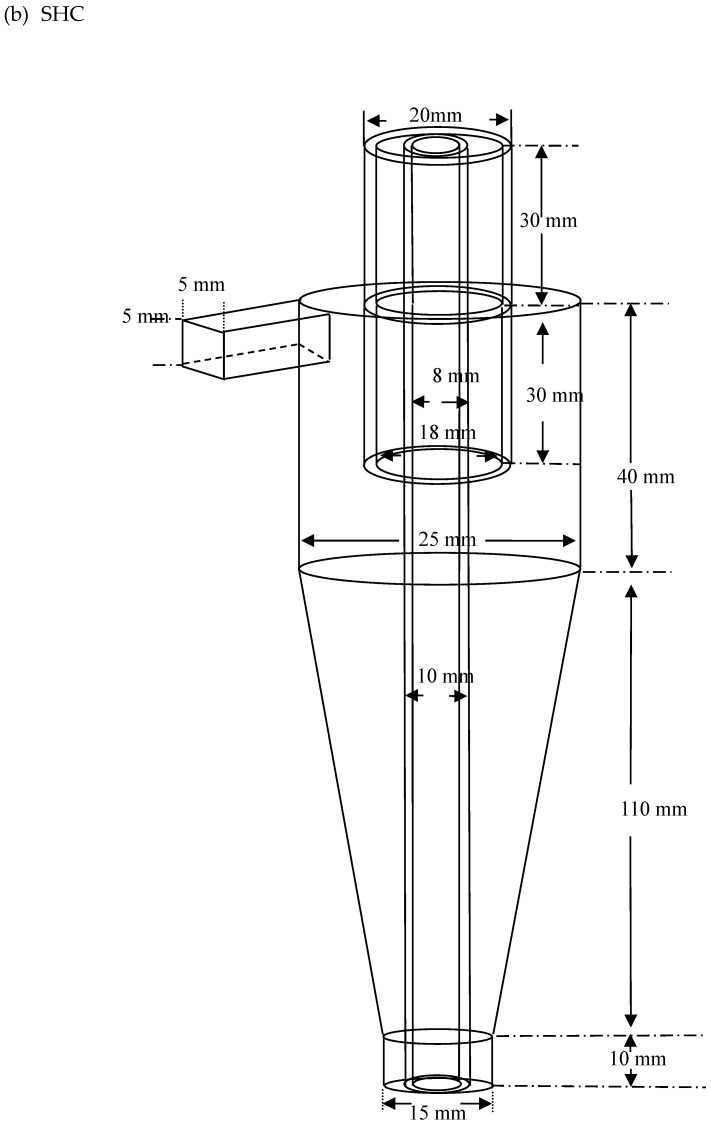

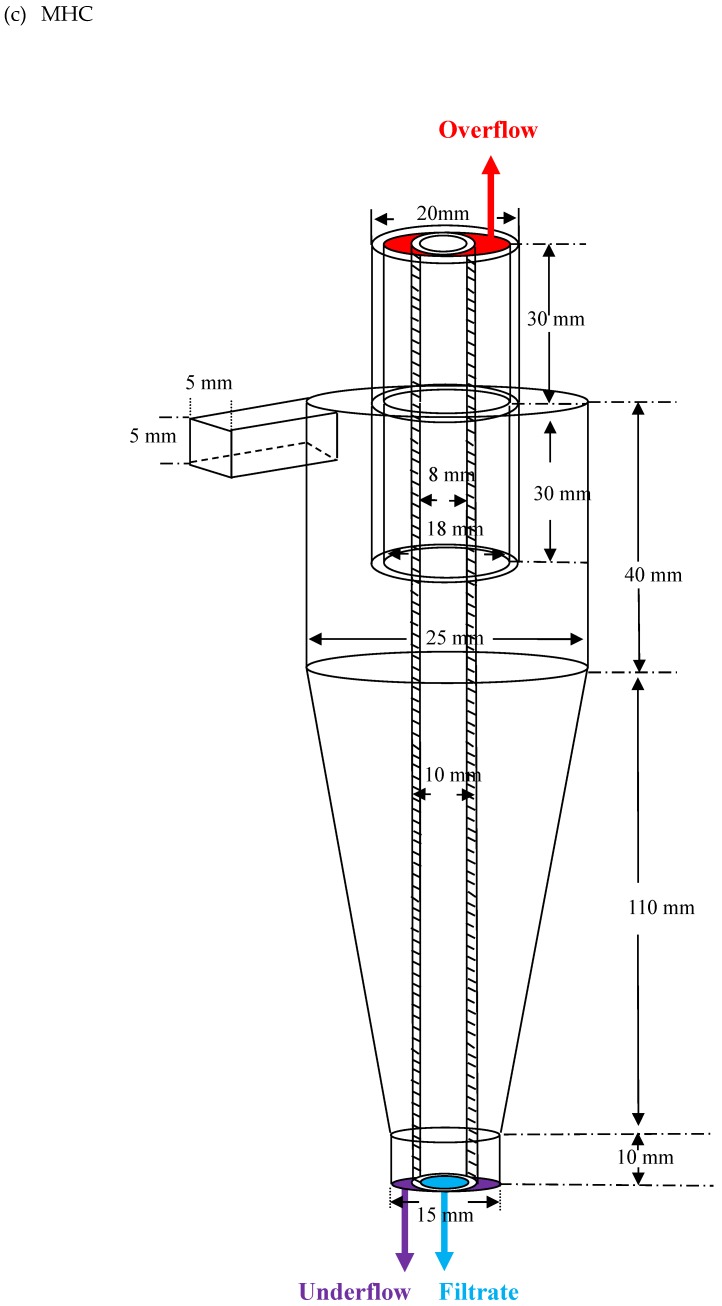
Hydrocyclone separator system geometry used for both computational fluid dynamics (CFD) simulations and experiments. (**a**) THC; (**b**) SHC; (**c**) MHC.

The three hydrocyclones types used in this study are presented in Figure 11. Figure 11a presents a THC. In Figure 11b, we used a 1 cm diameter solid cylinder inside the hydrocyclone to replace the original air core (SHC). The comparison of Figure 11c with Figure 11b indicates a 1 cm diameter solid cylinder and a 1 cm diameter tubular ceramic membrane (MHC). The conical section length of the hydrocyclone was 110 mm, and the angle between the vertical line was 2.6°; the diameter of the cylindrical section was 25 mm, with a length of 40 mm; the inlet size of hydrocyclone was 5 mm × 5 mm; and the overflow and underflow outlet diameters were 18 and 15 mm, respectively.

### 3.4. Calculating Methods

The flow pattern within the hydrocyclone was a turbulent flow, and the simulation method was LES. The air core was simulated using the volume of fluid model. Derivationally, the fluid volume fraction is decided by αv of each controlling body; the transport equation of air–water interface is defined in Equations (1) and (2):

(1) $$\frac{\partial }{\partial t}(\alpha_{w}\rho_{w}{\vec{v}}_{w})+\nabla •(\alpha_{w}\rho_{w}{\vec{v}}_{w}{\vec{v}}_{w})=-\alpha_{w}\nabla p+\nabla •{\bar{\bar{\tau }}}_{w}+\alpha_{w}\rho_{w}\vec{g}+\sum_{s=1}^{n}({\vec{R}}_{sw}+{\dot{m}}_{sw}{\vec{v}}_{sw})$$

(2) $$\frac{\partial }{\partial t}(\alpha_{s}\rho_{s}{\vec{v}}_{s})+\nabla •(\alpha_{s}\rho_{s}{\vec{v}}_{s}{\vec{v}}_{s})=-\alpha_{s}\nabla p+\nabla •{\bar{\bar{\tau }}}_{s}+\alpha_{s}\rho_{s}\vec{g}+\sum_{w=1}^{n}({\vec{R}}_{ws}+{\dot{m}}_{ws}{\vec{v}}_{ws})$$

The Lagrangian discrete phase model (DPM) follows the Euler–Lagrange approach. The fluid phase is considered a continuous phase and is governed by the time-averaged Navier–Stokes equation. However, the dispersed phase is solved by tracking trajectories of a large number of particles through the calculated flow field velocity. The dispersed phase can exchange momentum, mass, and energy with the continuous fluid phase.

**Figure 12:**
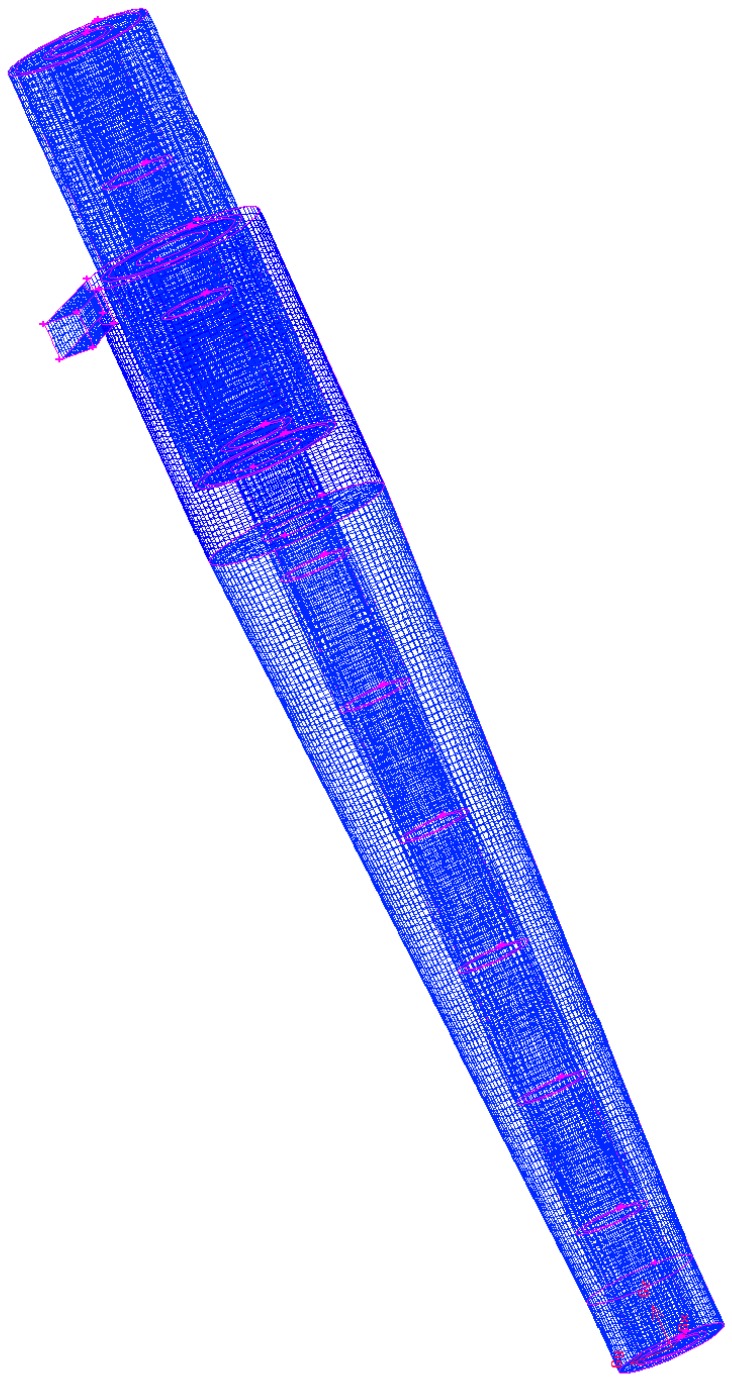
Hydrocyclone separator system mesh structure. The total number of computational cells was approximately 10^5^.

As depicted in Figure 12, the density of the mesh cell number 1,500,000 is presented. Mesh independence analysis indicates that it is suitable for simulating the optimum velocity value under a reasonable prediction computation time. Feeding fluid velocity is constant, and the boundary conditions are as follows:

(3) $$\vec{v}=\mathrm{constant}\;@\;\mathrm{inlet}\;\mathrm{pipe}$$

*P* = 0  @ underflow (4)

*P* = 0  @ overflow (5)

The outlet fluid was under atmospheric pressure, and therefore the overflow and underflow pressures of the flow were set to zero.

The hydrocyclone inner wall setting follows the no-slip boundary condition. Using governing Equations (1) and (2) as well as the relevant boundary condition Equations (3)–(5), CFD FLUENT 6.1 software (Fluent, NY, USA) calculates the flow field within the hydrocyclone. The pressure staggered option effectively predicted the high swirl flow within the hydrocyclone. The SIMPLE algorithm program, which uses a combination of continuous and momentum equations to derive pressure equation, was used in this simulation method. Calculations were implemented under the maximum relative error of 10^−4^ of the estimated fluid velocity.

## 4. Conclusions

In this study, silicon carbide particles were used to implement hydrocyclone experiments, and the simulation verification was calculated by using Fluent CFD software. Air core areas with metal cylinders and ceramic tubular membranes were compared, and the effects of the separation phenomena of different hydrocyclones were explored.

By inserting a tubular membrane, the resultant hydrocyclone has three outlets, namely the overflow dilute liquid, underflow concentrated liquid, and clear filtrate. This hydrocyclone can accomplish classification and filtration. The proposed hydrocyclone had a classification function equivalent to that of the conventional hydrocyclone. However, the overflow concentration was relatively dilute, the underflow concentration was relatively concentrated, and a clear filtrate was obtained.

## Figures and Tables

**Table 1:** Outlet concentration of various hydrocyclones.

Origin = 0.35 wt %				
	Pressure (Bar)	Over (wt %)	Under (wt %)	Filtration (wt %)
THC	2	0.324	0.405	-
	4	0.301	0.416	
SHC	2	0.295	0.423	-
	4	0.236	0.590	
MHC	2	0.321	0.821	0.004
	4	0.211	1.305	0.008
